# Strictures on the Usual Practice in Strangulated Hernia

**Published:** 1800-10

**Authors:** Edward Geoghegan

**Affiliations:** Member of the Royal College of Surgeons in Ireland. Dublin


### *Strictures on the usual Practice in Strangulated Hernia. By Mr.* Geoghegan.

To the Editors of the Medical and Physical Journal.

Gentlemen,

HAVING ſucceeded in reducing ſeveral inſtances of Strangulated Hernia by the taxis, where the ſymptoms were ſo alarming as to point out the neceſſity of immediate operation; I take the liberty of communicating, through the medium of your Journal, the practice which I purſued, and the principles upon which it was founded. I am the more deſirous of exciting the attention of practitioners to this d ſeaſe, from a conviction that the practice recommended by the lateſt writers of character on this ſubject, is productive of the moſt dangerous conſequences; Mr. Bell, whoſe work is in the hands of every ſurgeon, lays it down as a maxim, that we ought always to proceed to the operation, if after a few hours, two or three at the fartheſt, the remedies generally recommended do not prove effectual; and he does not think the operation, abſtractedly conſidered, attended with great danger. If we conſult the records of practice, ſurely we ſhall find a hoſt of evidence of the fallacy of this author's maxim; I am confident that the experience of every practitioner is in direct oppoſition to it; I have met with numberleſs inſtances, in which not only ſeveral hours, but days, have paſſed over before a reduction by the taxis could be effected in Strangulated Hernia, attended with ſingultus, vomiting of feculent matter, &c. and afterward the protruded parts were reduced without operation. Similar caſes have occurred to every experienced practitioner with whom I have communicated on the ſubject; and as to the queſtion, whether the operation is to be held as dangerous, abſtractedly conſidered, I aver aver that it is imminently ſo. We know that the danger attendant on every operation of conſequence, turns as much upon the peculiarity of the patient's conſtitution, independent of the diſeaſe which it is intended to relieve, as on any other circumſtance; the moſt minute conſideration therefore ſhould be given to every means of relieving, before it is had recourſe to in any caſe. In the preſent inſtance, a ſubject in full health is ſuddenly laid proſtrate, attacked by a painful affection, which exceedingly deranges the entire ſyſtem, not habituated to it by gradual ſuffering; a deſideratum preparatory to an operation. Experience inſtructs us he can ill bear any violence; his inteſtines are to be expoſed to the air, a tendinous part to be wounded, and air may be admitted into the cavity of the abdomen; beſide the effects which a ſlow diſſection muſt produce on a ſubject juſt removed from a ſtate of health, ſurely nothing can be more obvious than that ſuch caſes are in principle exceedingly dangerous. The inſtructions which are given by Mr. Bell and Mr. Pott, as to the method of reducing by the taxis, appear to me not only imperfect but injudicious; they direct that the patient being properly placed, "the ſurgeon is to graſp the inferior part of the hernia with one hand, and puſh upwards, whilſt he endeavours with the fingers of the other to puſh forward the parts at the ſuperior part of the tumor." Let us for a moment conſider the ſtate of the parts; a portion of inteſtine lies without an aperture, through which it is too large to paſs; the queſtion then ariſes, what occaſions its bulk? Surely, the nature of the part, the touch, and all the circumſtances of the caſe, clearly evince it to be flatus, and ſometimes together with excrement and an inflamed inteſtine, whoſe functions are ſo far deranged that it cannot act upon its natural contents, ſo as to move them in their ordinary courſe. The abdominal ring is in nowiſe concerned in the diſeaſe, only that as it is too ſmall to admit of the return of the inflamed and inflated parts, it unavoidably girts them; and nothing can be more abſurd than the idea of relaxing it, as from its ſtructure it muſt be paſſive. Let us ſuppoſe a ring fixed upon the neck of an inflated bladder, and that it is deſired that the bladder ſhould be paſſed through the ring, could any thing be more erroneous than to endeavour to puſh forward, and upwards, or any way, without removing the air which occaſioned the reſiſtance? The caſes are ſimilar, and nothing can be more obvious than that every effort ſhould made to leſſen the bulk of the hernia, and none to puſh it through the ring; it will paſs in of itſelf after the air has been extricated. It is obſerved by thoſe authors, that a guggling noiſe is always heard on the paſſing up of the inteſtine, which they merely mention, but do not inſiſt upon, as the great and principal circumſtance to be attended attended to in forming a juſt opinion of the mode of treatment. The mode of practice which I purſue, founded on the preceding principle, is as follows: I expoſe the entire body to the cold, naked, open the windows, &c. and apply cloths to the part, wet every ten minutes with a ſolution of muriated ammonia in vinegar and water; if ice can be procured, I prefer it. The patient generally complains much of the cold, and ſhivers, a deſirable circumſtance, as it induces ſuch a collapſe of the entire ſurface, as greatly to aſſiſt our intention of condenſing and expelling the air from its impriſonment, after about an hour ſpent in this way without touching the parts with the hands, or any effort by the taxis, as generally practiſed. Let the palms of the hands be applied to the ſides of the hernia, gently to preſs them towards each other, with a view to aſſiſt in propelling, not the inteſtine, but its contents, the air, &c. through the ring. In one inſtance, I ſucceeded in this way on the eighth day, in another on the ſixth, after the ſtrangulation had ſet in in both, attended with vomiting of feculent matter, hiccup, &c. Many efforts had been made in the uſual way of endeavouring to force the inteſtine upwards and forwards, which, I am of opinion, always exaſperates the ſymptoms. In another caſe, the diſeaſe had exiſted about twelve hours; the volume protruded was immenſe, and the ſymptoms ran exceedingly high; the patient was of a remarkably robuſt habit. After taking off about 20 ounces of blood, and having applied the cold ſolution for ſome time, I endeavoured with my hands to aſſiſt in extricating the air, but was unſucceſsful. Being obliged to go away, I directed that he ſhould remain naked, expoſed to the cold, and to perſevere in the cold fomentation. On my return, after half an hour, his rupture had diſappeared. He told me that he heard a noiſe as if wind had ruſhed out of it, and that it went up of itſelf. Some practitioners apprehend danger from the application of cold to a part ſo much inflamed; but the ſcrotum and ſac are ſecure intermediums to moderate its operations on the inteſtine; and experience proves that danger is not to be dreaded. From theſe conſiderations, I preſume, it is obvious that the directions given by moſt authors are not calculated to produce the deſired effect, particularly the operation of puſhing the inflamed and inflated parts through the ring, which is, *prima facie*, impoſſible; that the practice of condenſing and extricating the air, has a rational foundation; and that the ſole attention of the ſurgeon ought to be directed to that end.

I preſume it is underſtood that Bubonocele is the term of the diſeaſe I treat of.

EDWARD GEOGHEGAN,
Member of the Royal College of Surgeons in Ireland,

